# Protocol Reverse Analysis of Ethernet for Control Automation Technology Based on Sequence Alignment and Pearson Correlation Coefficient

**DOI:** 10.3390/s24247922

**Published:** 2024-12-11

**Authors:** Xiaopeng Wang, Yu Yao, Zhongwei Li, Changhe Su, Yunsong Tian

**Affiliations:** 1 School of Computer Science and Engineering, Northeastern University, Shenyang 110169, China; wangxiaopeng@nsfocus.com; 2 NSFOCUS Technologies Group Co., Ltd., Beijing 100089, China; 3 School of Electrical Engineering and Automation, Harbin Institute of Technology, Harbin 150001, China; lzw@hit.edu.cn (Z.L.); 22s106139@stu.hit.edu.cn (C.S.); 15034885038@163.com (Y.T.)

**Keywords:** protocol reverse engineering, sequence alignment, Pearson correlation coefficient, industrial protocol

## Abstract

This study presents a novel algorithm for protocol reverse analysis of EtherCAT. The algorithm combines sequence alignment and the Pearson correlation coefficient. We utilize value distribution statistics and the bit flip rate algorithm to effectively partition the protocol fields. We propose a semantics analysis method based on sequence alignment when HMI data and EtherCAT messages have a direct correlation. Conversely, for circumstances where there exists a decoding relationship between HMI data and EtherCAT messages, a semantic analysis method is proposed that employs the Pearson correlation coefficient. We completed a reverse analysis of the EtherCAT messages from an industrial robot system. By comparing the experiment results with the protocol description document, we validated the effectiveness of the method.

## 1. Introduction

The rapid advancement of computer network technology has led to the proliferation of private communication protocols with unfamiliar formats on the internet. These protocols pose significant challenges in analyzing network security. Researchers have introduced the concept of protocol reverse engineering to address this issue. Protocol reverse engineering involves extracting protocol semantic information from network packets and communication programs, even without prior knowledge [1]. This technology assists professionals in analyzing protocol specifications and has found comprehensive implementation in various network security sectors, including the development of firewalls, intrusion detection systems and fuzzing tests.

Significant advancements have been made in the development of reverse engineering approaches applied to conventional internet protocols. Initially, reverse engineering was performed through manual analysis, which was time-consuming and prone to errors. For instance, the renowned Samba project took 12 years to extract specifications for SMB protocols [2]. Since then, researchers have developed various automated tools for protocol reverse engineering, such as PIP [3], Netzob [4], and more recently, deep learning-based techniques [5]. These tools demonstrate the progress in internet protocol reverse engineering.

The security of industrial control networks has received significant attention due to the increasing integration of internet technologies with industrial control systems (ICS) in recent years [6]. Industrial control systems rely on numerous private industrial protocols developed by equipment manufacturers. However, these protocols were initially designed without considering security, making them vulnerable to network security vulnerabilities that can be exploited by attackers, potentially leading to severe consequences [7].

Industrial robotics systems are vital components of industrial control systems, which have a crucial role in manufacturing. If there are cyberattacks on the cyber manufacturing system, it can lead to significant and irreversible impacts. However, analyzing the cybersecurity of industrial robotics systems becomes challenging due to the use of private protocols. One potential solution to address this issue is the utilization of protocol reverse engineering techniques. It is important to note that there are differences between industrial protocols and conventional network protocols.

Compared with traditional network protocols, industrial protocols focus on the formatting and encoding of messages that contain operational instructions and control information. Analyzing operational instructions requires adjusting conventional protocol reverse engineering techniques. Therefore, it is essential to research reverse engineering techniques specifically designed for industrial protocols. Wei et al. implemented reverse parsing of industrial protocols using static binary code analysis tools that are based on dynamic taint analysis [8]. In [9], the authors introduced AutoBoundary, a method that constructs a memory propagation tree from contextual information obtained during program execution. This method automates the identification and analysis of protocol field boundary information. Based on program analysis, these techniques enable a precise analysis of the semantics of industrial control protocols.

However, the implementation of these methods is challenging as it requires researchers to possess a deep understanding of industrial control procedures and may impact the stability of the industrial control system. Wang et al. proposed a multi-stage integrated reverse analysis method, known as staged identification, to enhance the accuracy of field segmentation by leveraging the characteristics of industrial protocols [10]. In another study [11], an improved voting expert algorithm was employed to partition the boundaries of industrial protocol fields and infer their types. Additionally, the messages were classified based on the field types, leading to precise field partitioning. Although these methods are based on network traffic analysis, they require more specificity to achieve semantic inference that is relevant to specific industrial scenarios.

Previous studies have achieved certain results in protocol field segmentation and semantic analyses. However, significant limitations remain, particularly regarding the accuracy of protocol field semantic inference and implementation complexity. Existing methods often rely on a rule-based analysis approach or pattern-based analysis approach. Although these methods perform basic protocol parsing tasks, they frequently fail to accurately infer the meanings of protocol fields in specific industrial environments, especially under complex protocol scenarios. The limitations of these approaches are reflected in three main aspects: First, many traditional approaches depend on static rules and predefined templates, limiting their adaptability to dynamically changing or unseen protocol variants. Second, existing semantic inference methods frequently overlook the deep connections between protocol fields and actual control data, leading to less precise analyses, particularly in complex industrial control systems. Lastly, while some methods attempt to incorporate machine learning or deep learning techniques, these approaches are challenging to implement in practical applications due to the lack of extensive labeled data. Their generalization capability and robustness also remain unverified.

To address these issues, this paper proposes a novel EtherCAT protocol reverse analysis method, aiming to overcome the limitations of traditional methods. Unlike conventional approaches, the proposed algorithm not only employs binary sequence analysis but also integrates Human–Machine Interface (HMI) data from industrial robot systems to significantly improve protocol field semantic analysis accuracy. Specifically, the proposed algorithm selects the appropriate semantic analysis technique based on the correlation between HMI data and EtherCAT messages: when HMI data are directly correlated with EtherCAT messages, a semantic analysis algorithm based on sequence alignment is used; when a decoding relationship exists, an algorithm based on Pearson’s correlation coefficient is applied. This innovative approach combines field data (HMI data) with protocol analysis, resulting in more accurate field semantic inferences. It is relatively easy to implement in practical applications. Compared to existing methods, the proposed algorithm offers superior accuracy and adaptability, particularly for dynamically evolving industrial protocols. Furthermore, it requires neither complex manual rules nor large amounts of labeled data, enhancing its practicality and reliability.

The remaining part of the paper is organized as follows: Section 2 presents the design of a reverse analysis method for the EtherCAT protocol, which is based on sequence alignment and the Pearson correlation coefficient. In Section 3, we describe industrial scenarios and the process of data acquisition. The effectiveness of the reverse analysis method was verified through experiments presented in Section 4. Finally, Section 5 provides the conclusion and future outlook.

## 2. Methodology

**Figure 1:**
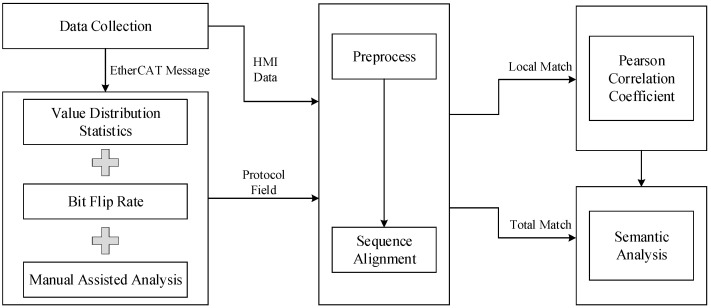
EtherCAT protocol reverse analysis framework.

This section presents a reverse analysis method for EtherCAT protocols using sequence alignment and the Pearson correlation coefficient. The method framework is illustrated in Figure 1. We first analyze the characteristics of EtherCAT and the security requirements for applications in industrial scenarios. For the captured EtherCAT messages, we employ the method of value distribution statistics to divide messages into multi-value fields, fixed fields and highly variable fields. Subsequently, we utilize statistics of the bit flip rate to further divide the highly variable fields into signal fields, counter fields and checksum fields. Manual analysis is also incorporated to complete the field division of the message. Moreover, we exploit the direct correlation between HMI data and messages, which leads to the proposal of a semantics analysis method based on sequence comparison. Finally, we introduce a protocol semantics analysis method based on the Pearson correlation coefficient to analyze the semantic relationship between the HMI data and messages in the EtherCAT protocol, taking into account their linear correlation.

### 2.1. EtherCAT Protocol Analysis

Ethernet for Control Automation Technology (EtherCAT) is an efficient industrial Ethernet communication protocol, particularly suited for real-time control systems. Compared to traditional Ethernet protocols, EtherCAT offers significant advantages in data transmission speed and real-time performance. The EtherCAT protocol supports high-speed and precise time synchronization, making it ideal for applications with high real-time requirements, such as industrial automation, robotics control, and measurement equipment. In an EtherCAT network, the master node controls the slave nodes to complete real-time data acquisition and control tasks. Each slave device not only receives data but can also transmit data directly to the next node, effectively reducing data transmission delays and bandwidth usage.

Although EtherCAT excels in transmission speed and synchronization, its data packets often contain a large amount of application-specific control information, which may have different meanings in various industrial scenarios. To effectively understand and analyze the data within the EtherCAT protocol, semantic analysis is required to extract specific control instructions, sensor data, device status, and other information. This enables the identification of potential malicious packets or non-compliant protocol usage behaviors during network attack prevention.

### 2.2. Field Segmentation Based on Value Distribution Statistics

Sequence alignment and Pearson’s correlation coefficient have been widely used in protocol reverse engineering; however, sequence alignment methods rely heavily on the sequential consistency of the data and are more sensitive to the noise and variation of the data, and therefore may not yield accurate results when dealing with protocol data with large variations or noise. The Pearson correlation coefficient, on the other hand, is unable to deal with non-linear relationships and does not provide a precise semantic resolution between fields. The proposed method combines the advantages of sequence alignment and Pearson’s correlation coefficient in protocol semantic analysis, using Pearson’s correlation coefficient to supplement the sequence matching method in dealing with different variation patterns, and strengthening the accuracy and stability of protocol semantic analysis by taking into account the sequential similarity and potential correlation between fields, especially in the face of the complexity or irregularity of the industrial protocols, which shows superior performance.

To improve the accuracy and efficiency of semantic analysis, it is crucial to divide the message fields before conducting the protocol semantic analysis. The division process starts by using value distribution statistics to track the changes in the value of each byte in the sub-message data over a specific time period. We can classify the fields into three categories: fixed fields, multi-value fields and highly variable fields [9,10].

Fixed fields have constant values and remain unchanged over time. Multi-value fields have a limited number of values. Highly variable fields exhibit a more widely dispersed distribution of values and encompass a larger number of different types.

The focus of field segmentation is to analyze multi-value fields and highly variable fields. The extraction of these fields can be achieved through value distribution statistics. Subsequently, the bit flip rate feature is used to further facilitate the segmentation of multi-valued and highly variable fields based on the previous results.

### 2.3. Field Segmentation Based on Bit Flip Rate Feature

#### 2.3.1. Definition of Bit Flip Rate

The number of bit flips of bit *j* in the sub-message *b* over a period of time *t* (*N_t_*)can be defined as shown in Equation (1) [12] when representing the EtherCAT message as a binary sequence, where *k* is the length of *b*, and *n* is the number of *b* in *t*.

(1) $$N_{j}=\sum_{i=2}^{n}1,\forall j\in [1,k]\cap b_{i,j}\neq b_{i-1,j}$$

The bit flip rate of bit *j* over time *t* can be computed by Equation (2), where the definitions of *N_j_*, *n* are as described for Equation (1).

(2) $$R_{j}=\frac{N_{j}}{n}$$

The bit flip rate feature of different fields is shown in Table 1.

#### 2.3.2. EtherCAT Message Field Segmentation Algorithm

**Figure 2:**
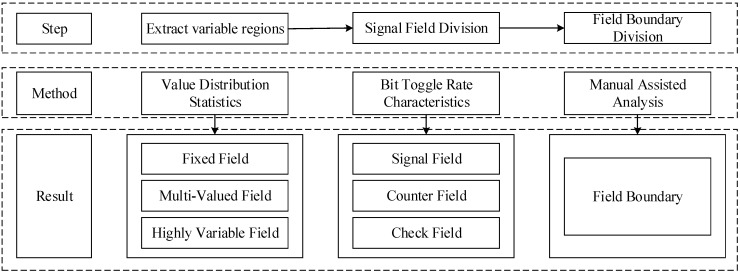
EtherCAT message field segmentation algorithm.

The proposed EtherCAT message field segmentation algorithm, as shown in Figure 2, consists of three phases:

(i) Extracting Variable Regions: The EtherCAT messages are divided into the invariant region (fixed fields) and the variable region (multi-value and highly variable fields) based on value distribution statistics.
(ii) Signal Field Division: The field types in the variable region are further examined through the analysis of the bit flip rate feature. Choosing an excessively high ratio may lead to over-segmentation, causing some practically related fields to be split into multiple smaller parts, thus increasing the complexity and instability of data parsing. Conversely, selecting too low a ratio may result in insufficient segmentation, making it difficult to effectively distinguish the boundaries of different fields in the protocol, which in turn affects the accuracy of semantic analysis. Considering that the bits in an EtherCAT frame only represent two values, the bit flip rate ratio of 0.5, derived from the statistical distribution, is appropriate. Preliminary field boundaries are identified when the quotient of the bit flip rate between adjacent high-order bits and adjacent low-order bits is less than 0.5.
(iii) Field Boundary Division: The bit flip rate algorithm’s output results are analyzed for reasonability and appropriate adjustments are made based on manual experience.

### 2.4. Semantic Analysis Based on Sequence Alignment

The concept of sequence alignment originated in the field of bioinformatics. It involves arranging and aligning two or more sequences in a specific manner to compare corresponding positions and determine if the values at each position are the same. This process helps calculate the local or overall similarity between sequences. Beddoe was one of the pioneers in applying sequence alignment to protocol reverse engineering. He used string alignment to analyze the start and end positions of HTTP protocol fields [3]. In this paper, we utilize the Smith–Waterman algorithm (SW), known for its flexibility and high accuracy, to analyze the semantics of protocol fields.

The Smith–Waterman (SW) algorithm is a local sequence alignment algorithm used to identify the most similar local sequences between two given sequences. It compares corresponding characters in locally similar sequences to calculate alignment scores and generates a scoring matrix based on these scores. The algorithm determines the optimal alignment between sequences by performing backtracking from the cell with the highest score in the matrix.

Assuming the existence of sequences $A=[a_{1},a_{2},\ldots ,a_{n}]$ and $B=[b_{1},b_{2},\ldots ,b_{m}]$, the Smith–Waterman algorithm consists of five steps:

(1) Construct the score matrix H: Represent the elements of two sequences A and B in rows and columns, respectively and initialize the matrix.
(2) Calculate the scores of the cells in the matrix H(i,j).

(3) $$H(i,j)=\max\left\{\begin{array}{l} H(i-1,j)-K\\ H(i,j-1),-K\\ H(i-1,j-1)+S(a_{i},b_{j})\\ 0 \end{array}\right.$$

(4) $$S(a_{i},b_{j})=\left\{\begin{array}{l} const_{1},(a_{i}=b_{j})\\ const_{2},(a_{i}\neq b_{j}) \end{array}\right.$$

where $S(a_{i},b_{j})$ is the similarity score between elements of $a_{i}$ and $b_{j}$, and $const_{1}$ and $const_{2}$ are two predefined constant values representing the similarity scores for matching and non-matching characters, respectively. H is the score matrix and K is the penalty score for a gap (empty position) and it can be set appropriately according to the requirements.

**Figure 3:**
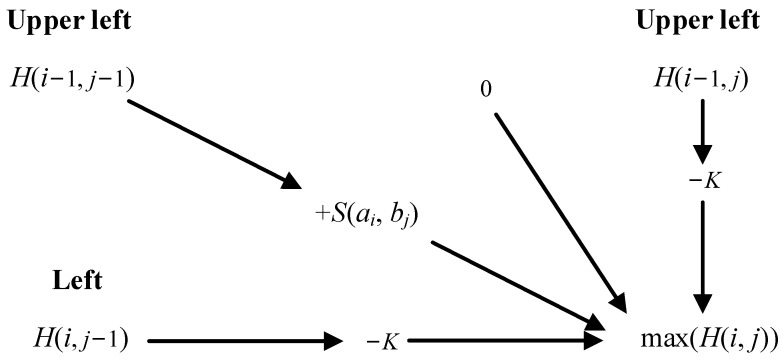
The calculation method of H(i,j).

The calculation method of H(i,j) is shown in Figure 3.

(3) Backtrack from the entry with the highest score in matrix H until reaching a cell with a value of 0.
(4) Extract the matching results according to the backtrack path.
(5) Obtain all matching sequences and the algorithm terminates.

**Figure 4:**
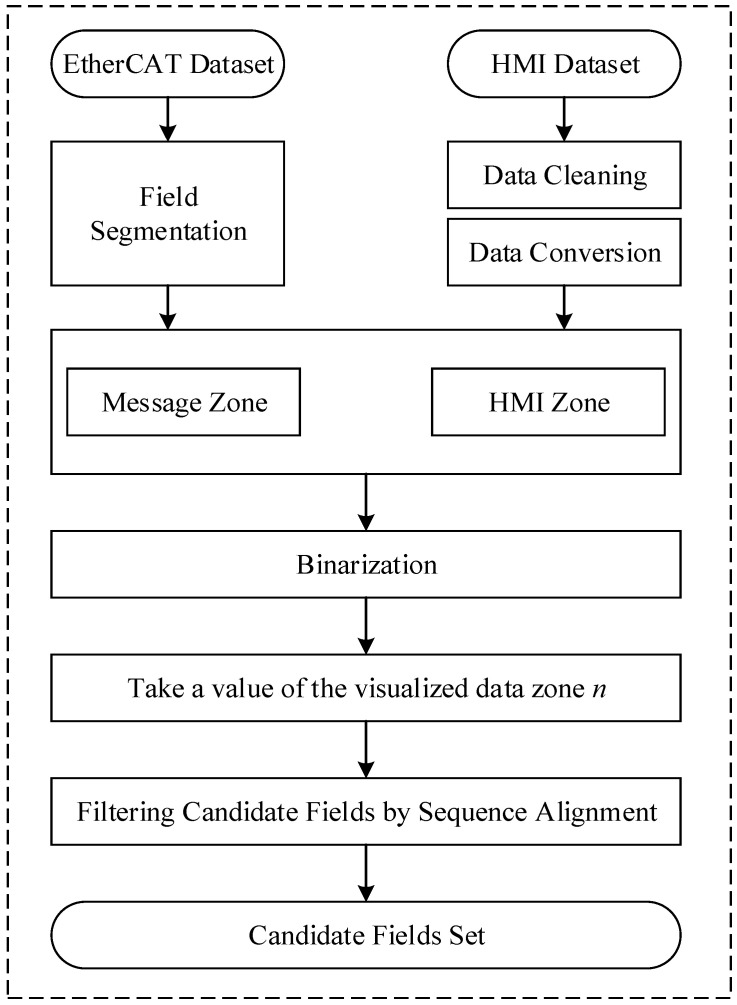
Semantic analysis framework based on sequence alignment.

This paper proposes a semantic analysis framework based on sequence alignment, illustrated in Figure 4. The framework mainly consists of four steps:

(1) Identifying visual data from various regions and applying normalization techniques to prepare it for subsequent semantic analysis.
(2) Select a specific value from data region *n* on the HMI interface and conduct a matching analysis with the values collected from the message fields during the same period. If the data content in the collected messages matches the data region *n*, the corresponding message field will be considered a candidate region field. This step will generate a series of candidate regional fields.
(3) Retrieve a value from data region *n* on the HMI interface that differs from the previously selected values. Next, correlate the selected visual data with the candidate region fields from the EtherCAT messages. Any candidate region field with a poor matching effect will be excluded. If there are multiple remaining candidate region fields, repeat this step.
(4) Check if all visual data regions match the message fields. If there are still regions with unresolved semantics, continue the analysis. If all regions have been successfully resolved, stop the analysis.

### 2.5. Semantic Analysis Based on Pearson Correlation Coefficient

The sequence alignment algorithm can accurately infer the protocol semantics when there exists a direct correspondence between the HMI data and message fields. However, the sequence alignment algorithm may not accurately analyze the semantics if there is a decoding relationship between the HMI data and message fields. This situation occurs when the HMI data are not directly converted from the message to decimal representation for display but involves a specific decoding relationship. Previous studies suggest that this decoding relationship may be linear. We incorporate the Pearson correlation coefficient into the protocol for reverse engineering. The formula for calculating the Pearson coefficient is provided as (5):

(5) $$\rho_{X,Y}=\frac{\mathrm{cov}(X,Y)}{\sigma_{x}\sigma_{Y}}=\frac{E[(X-\mu x)(Y-\mu Y)]}{\sigma_{x}\sigma_{Y}}$$

when there is no direct correspondence between HMI data and message fields, we can set $m_{s}$, $t_{s}$, and $d_{s}$ as the resolution, offset, and actual value, respectively. The decoding relationship is represented by Equation (6):

(6) $$d_{s}=m_{s}\cdot r_{s}+t_{s}$$

According to Equation (6), it is clear that the relationship between $r_{s}$ and $d_{s}$ is linear. The actual value represented by $d_{s}$ can be obtained from HMI. The original value of the message field $r_{s}$ can be obtained by collecting EtherCAT messages.

Let *a* and *b* represent the EtherCAT message fields and HMI data, respectively. $m_{a}(i)$ and $m_{b}(i)$ are the *i*-th message field and the HMI data collected for a period, respectively. n is the number of datapoints used for the calculation within that period. $\mu_{a}$ and $\mu_{b}$ are the means of EtherCAT message data and HMI data, respectively. The correlation between them can be expressed using the Pearson correlation coefficient, denoted by ρ:

(7) $$\rho =\frac{\sum_{i=1}^{n}\left(m_{a}(i)-\mu_{a})(m_{b}(i)-\mu_{b}\right)}{\sqrt{{\sum_{i=1}^{n}\left(m_{a}(i)-\mu_{a}\right)}^{2}{\sum_{i=1}^{n}\left(m_{b}(i)-\mu_{b}\right)}^{2}}}$$

The Pearson correlation coefficient ranges from −1 to 1. When the absolute value of ρ is closer to 1, it indicates a stronger correlation. A Pearson correlation coefficient closer to 1 suggests a strong positive correlation between the HMI data and message field, thereby confirming the semantics of the field.

## 3. Scenarios of EtherCAT Application

**Figure 5:**
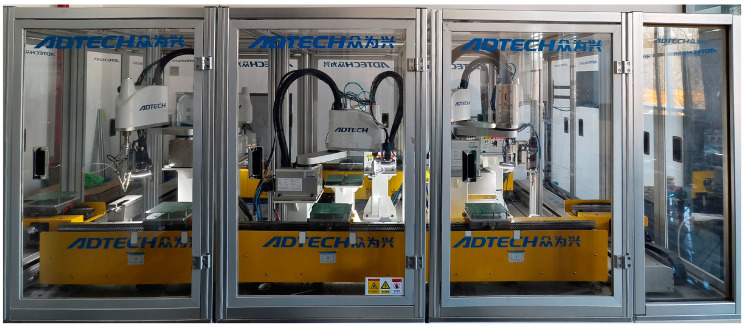
EtherCAT industrial robot system.

Figure 5 illustrates the EtherCAT industrial robot system used in this paper for data collection.

**Figure 6:**
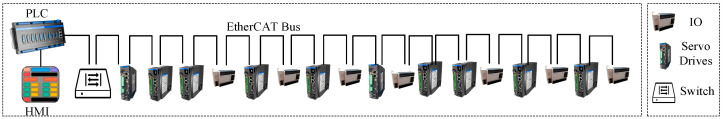
EtherCAT network connection diagram.

Figure 6 illustrates the EtherCAT network connection diagram. The servo drives receive control commands from PLC and provides feedback on the collected status information to the PLC. The PLC decodes the message data and displays the system status information on the HMI. We utilize the Wireshark tool to capture EtherCAT messages at the switch and collect processing data from the HMI interface.

## 4. Experimental Results and Discussion

**Figure 7:**
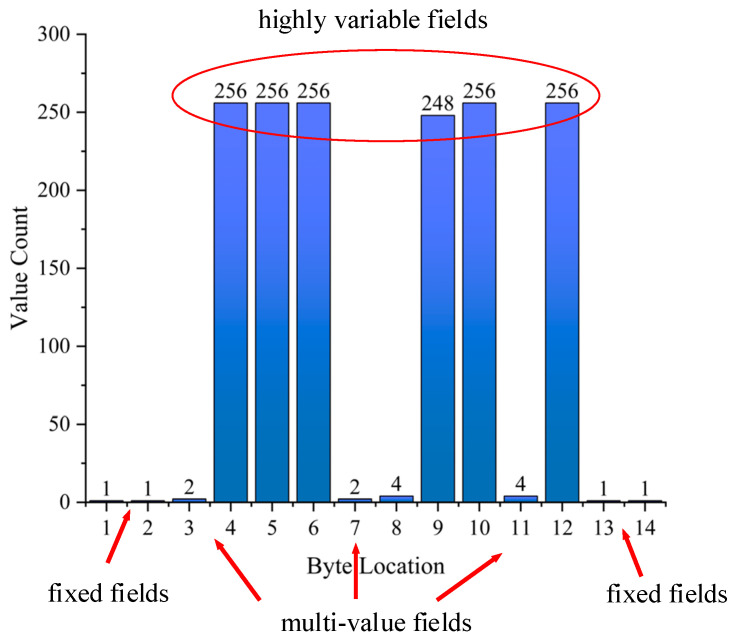
Results of EtherCAT message value distribution statistics.

We conducted a statistical analysis on a specific EtherCAT sub-message consisting of 14 bytes. The purpose was to examine the distribution of values in each byte. Based on the statistical results, we categorized the sub-message into three parts: fixed fields, highly variable fields, and multi-value fields. The fixed fields had only one value, while the highly variable fields had more than ten values. The multi-value fields, on the other hand, had fewer than ten values. Figure 7 presents the value distribution statistics for each byte, with the fixed fields identified as [1,2,12,13], the multi-value fields as [3,7,8,11], and the highly variable fields as [4,5,6,9,10,14].

**Figure 8:**
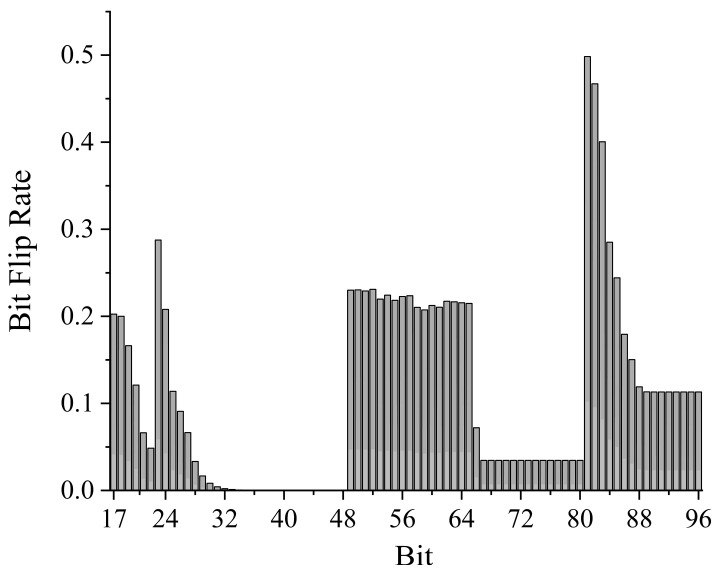
Results of EtherCAT message bit flip rates feature.

We calculated the bit flip rate for the multi-value and highly variable fields (i.e., from the 3rd to the 12th byte) with Equation (1). The results are shown in Figure 8. It is evident that the bit flip rates between the adjacent pairs of bits (17–22, 23–48, 49–80, and 81–96) exhibit minimal differences. This observation aligns with the characteristics of bit flip rates in signal fields as described in Section 2.3.1. Therefore, we categorize these regions as signal fields.

Further analysis is necessary to evaluate the validity of industrial control message fields, as they are typically organized on a byte basis. In this paper, we investigated the linear relationship between the difference between adjacent message bytes and the number of messages to determine if a byte belongs to the same field. Equation (8) presents the formula for assessing the linear relationship:

(8) $$\left\{\begin{array}{l} BJC_{i}=BZ_{i}-BZ_{i-1}\\ AVG_{bj}=\frac{\sum_{i=1}^{k}BJC_{i}}{k}\\ Y_{bj}=\frac{count(0.9AVG_{bj}<BJC_{i}<1.1AVG_{bj})}{k} \end{array}\right.$$

where $BZ_{i}$ represents the specific value of the *i*-th byte collected from the message, $BJZ_{i}$ represents the byte difference between the *i*-th and the (*i* − 1)-th collected messages, k represents the number of difference values, $AVG_{bj}$ denotes the average value of the differences, and $Y_{bj}$ is the parameter representing the uniformity of the field value distribution. If $Y_{bj}$ is closer to 1, it indicates a stronger linear relationship between the byte value distribution and the number of message bars.

**Figure 9:**
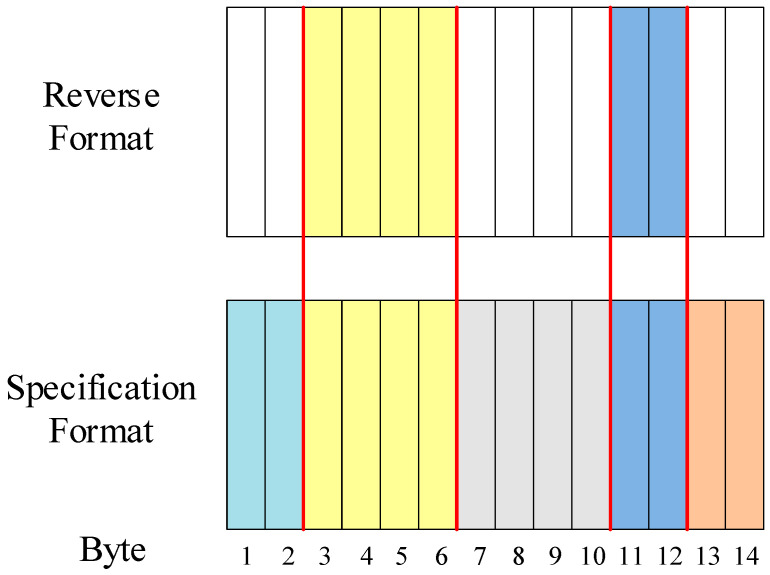
Results of EtherCAT message field division.

Based on statistical experience in protocol reverse engineering, the threshold for the parameter $Y_{bj}$ representing the uniformity of the field value distribution is set to 0.6. If $Y_{bj}$ is greater than 0.6, this indicates a strong linear relationship between the byte value distribution and the number of transmitted messages. In such cases, the byte is treated as a whole for field partitioning. In this study, the uniformity parameter for the relevant byte was calculated to be 0.9021. As a result, the bit flip rate results were adjusted by considering the byte as a whole. The modified message field partitioning results are presented in Figure 9, which align with the application layer protocol specifications, demonstrating the effectiveness of the protocol field partitioning algorithm proposed in this research.

Based on the results of field partitioning, the signal fields were identified as candidate fields. The sequence alignment algorithm was employed to verify further the correspondence between the HMI data and the candidate fields.

**Figure 10:**
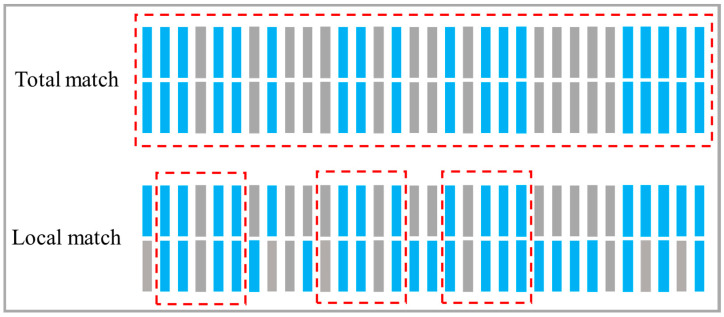
Example of sequence alignment matching results.

Firstly, an analysis was conducted on the possible signal fields based on the format of the HMI data. The collected HMI data consists of decimal numbers of 8 bits or more, which is difficult to express in 2 bytes. Therefore, Signal Field III was excluded and it is inferred to be located in the 4-byte-long Signal Field I and Signal Field II. Both HMI data and candidate fields were converted into a unified 32-bit binary format. Next, a sequence alignment was performed to analyze the field semantics. The output results of the sequence alignment algorithm, including complete matches and partial matches, are shown in Figure 10. A complete match indicates a direct correspondence between HMI data and EtherCAT messages, confirming that the meaning represented by the HMI data is the field semantics. In cases where only partial matches exist, this suggests that they do not correspond directly, and further assessment using the Pearson correlation coefficient is required.

We assigned a score of 1 for matching two characters in the sequence and a score of −1 for non-matching characters. Additionally, we set the penalty score for a gap to −0.5. The output results were determined based on these matching scores.

(9) $$result=\left\{\begin{array}{l} total\;match,\;score=32\\ local\;match,\;score<32 \end{array}\right.$$

**Figure 11:**
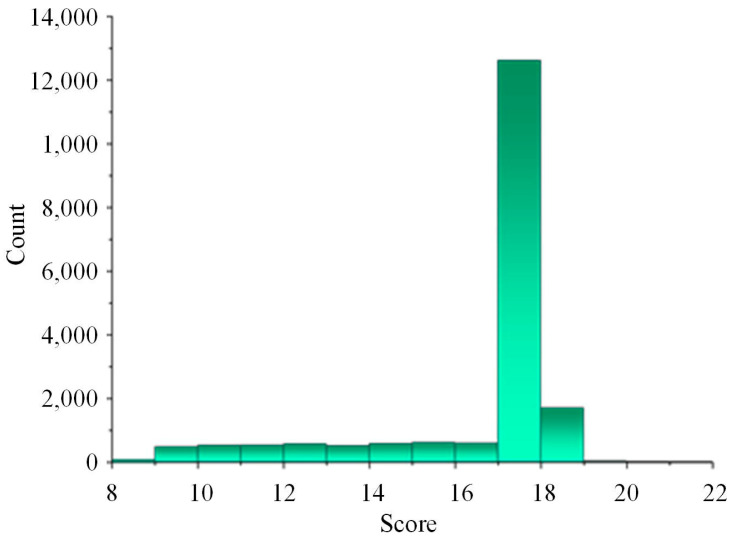
Match Score Statistics of Sequence Alignment.

According to Figure 11, all sequence matching scores are below 32, indicating that there are no complete matches. However, there are partial matches, which suggests that the protocol semantic analysis algorithm based on sequence alignment did not identify appropriate candidate regions for field semantics. This implies that EtherCAT messages cannot be directly displayed on the HMI interface through simple number conversions, and further semantic analysis using the Pearson correlation coefficient is necessary.

**Figure 12:**
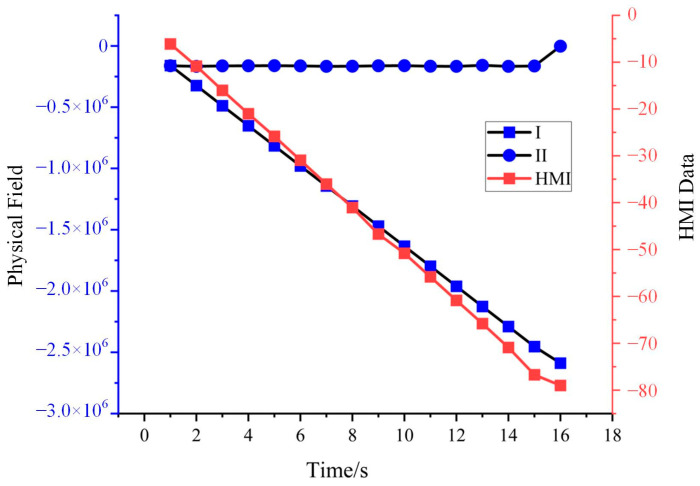
Amount of change in HMI data and EtherCAT data.

For each cycle of EtherCAT messages and HMI data, the changes in the signal field values and HMI values are calculated at 1 s intervals. The trend of these changes is depicted in Figure 12. The horizontal axis represents the unit time, while the vertical axis represents the change values. Figure 12 demonstrates a linear relationship between Signal Field I and the HMI data.

**Figure 13:**
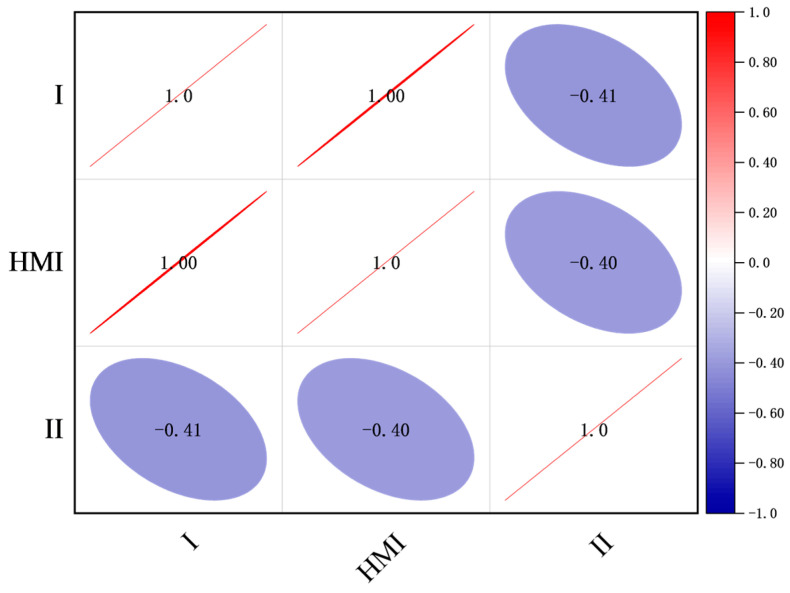
Pearson correlation coefficient calculations.

Figure 13 shows that the Pearson correlation coefficient between Signal Field I and HMI data is nearly 1, suggesting a robust linear relationship. Conversely, the Pearson correlation coefficient between Signal Field II and HMI data is −0.4, indicating a weaker correlation. These findings allow us to conclude that Signal Field I aligns with the HMI data, thus confirming the field’s semantics.

To verify the accuracy and running efficiency of the proposed protocol semantic analysis method, this paper compared the sequence alignment and sequence correlation analysis components of the proposed method with several classical algorithms. The input data for this comparison includes Signal Segment 1 and HMI data. The algorithms were evaluated based on sequence alignment accuracy, sequence alignment running time, sequence correlation metrics, and sequence correlation running time. The results are shown in Table 2. It can be observed that, in the sequence alignment step, Fuzzy String Matching yields the best matching performance but has a relatively longer running time. In comparison, our method outperforms Needleman–Wunsch and Dynamic Time Warping in terms of matching accuracy, while also being more efficient than Fuzzy String Matching and Dynamic Time Warping in terms of execution time. In the sequence correlation analysis step, the similarity measure results from all algorithms are generally consistent, but the proposed method excels in running efficiency, which is particularly advantageous for real-time Ethernet protocols like EtherCAT.

## 5. Conclusions

This paper proposes a protocol reverse analysis method for EtherCAT based on sequence alignment and Pearson correlation coefficient. We employ HMI data in protocol reverse engineering to enhance the accuracy of industrial control protocol semantic analysis. By collecting EtherCAT messages from an industrial robot system and utilizing value distribution statistics and bit flip rate characteristics, we achieve accurate partitioning of message fields. Sequence alignment analysis is used to determine the direct correlation between EtherCAT signal fields and HMI data, while the Pearson correlation coefficient is employed to explore the linear relationship between HMI data and EtherCAT signal fields, achieving precise semantic analysis. The research results demonstrated that the proposed EtherCAT protocol semantic analysis method achieves accurate field partitioning and semantic inference, effectively addressing the limitations of traditional reverse engineering techniques developed for internet protocols, which are not applicable to industrial protocols.

With the deep development of industrial internet technology, Ethernet-based industrial control protocols such as EtherCAT, Modbus TCP, EtherNet/IP, and PROFINET are widely applied in industrial control systems. The method proposed in this paper is not only applicable to the EtherCAT protocol but also has the potential for semantic analysis of the aforementioned protocols. Although their data structures and communication mechanisms differ, they share the core requirements of Ethernet-based communication architecture and field semantic analysis. The data frames of these protocols typically include clear field delimiters, enabling the binary sequence analysis module of our method to effectively perform field segmentation. Therefore, in future work, we will focus on the specific optimization of our method for these industrial internet protocols to enhance the general applicability of the proposed semantic analysis method in the industrial control domain. Furthermore, in future work, we will further analyze other fields in EtherCAT and explore their semantics by integrating the system operating environment and business information.

## Figures and Tables

**Table 1:** Bit flip rate features of different fields.

Field Type	Bit Flip Rate Features
Fixed fields	No change occurs and the bit flip rate is 0.
Multi-value fields	Uncertainty in bit flip rates and associated with the pattern of events.
Signal fields	Small difference in bit flip rate between two consecutive binary bits.
Counter fields	The bit flip rate is highest for the lowest bit, and the bit flip rate for the high bit between two neighboring bits is twice as low as that for the low bit.
Check fields	The bit flip rate obeys a normal probability distribution centered at 0.5.

**Table 2:** Algorithm Performance Comparison.

Sequence Alignment			Sequence Relation		
Algorithm	Value	Running Time (s)	Algorithm	Value	Running Time (s)
Ours	18.34	1.452	Ours	1	0.074
Fuzzy String Matching	22.30	1.984	Mutual Information	1	0.165
Needleman–Wunsch	12.63	1.091	Kendall’s Tau	1	0.161
Dynamic Time Warping	4.38	1.594	Spearman Rank Correlation	1	0.198

## Data Availability

The data can be shared on request.
